# Results of a German wide survey towards current surgical approach in early stage cervical cancer NOGGO MONITOR 11

**DOI:** 10.1038/s41598-021-89071-0

**Published:** 2021-05-07

**Authors:** Robert Armbrust, Frank Chen, Rolf Richter, Mustafa Zela Muallem, Alexander Mustea, Bernd Holthaus, Jalid Sehouli

**Affiliations:** 1 Department of Gynecology with Center for Oncological Surgery, Corporate Member of Freie Universität Berlin, Humboldt-Universität zu Berlin, and Berlin Institute of Health, Berlin, Virchow Campus Clinic, Charité Medical University, Augustenburger Platz 1, 13353 Berlin, Germany; 2 Humboldt-Universität Zu Berlin, and Berlin Institute of Health, Freie Universität Berlin, Berlin, Germany; 3 Department of Gynecology, University of Bonn, Bonn, Germany; 4 Department of Gynecology, Kardinal Von Galen Kliniken Damme, Working Group Gynecological Endoscopy, Damme, Germany

**Keywords:** Oncology, Surgical oncology

## Abstract

Minimally invasive surgery (MIS) has become the standard approach in early stage cervical cancer (ECC). However, the recently published “LACC” trial and even others could show inferior PFS and OS of MIS compared to open radical hysterectomy. The results led to a widespread debate about the best surgical approach in ECC. The present survey aimed to get first insights after publication. NOGGO and AGE conducted a nationwide digital survey among 186 Gynecological Cancer Centers. Descriptive statistics and t-tests were performed using SPSS. A majority of the centers were of high expertise and/or experience in treatment of ECC and were highly aware of the LACC trial results. Trial quality and scientific value were rated as very good/good. However, still 40% would not change the standard of care to open surgery. Centers with higher volume and participating in clinical trials were more likely to change. This survey represents insights after the surprising results of recently published trials towards the surgical approach of ECC. There still seems to be a high need of future trials and possible explanations for the unexpected worse outcomes in the MIS group.

## Introduction

The surgical approach to early stage cervical cancer has undergone significant evolutions and adjustments over the past decades. Different classifications of radical hysterectomies were developed^1^ and the surgical techniques themselves were redefined e.g. nerve-sparing techniques^2^ or the introduction of sentinel node lymphadenectomy^3^. The minimally invasive surgery (MIS) has become the standard surgical treatment of choice in Germany and Europe and was recommended by many guidelines (e.g., ESGO guidelines^4^). However, in February 2018 the results of the so called LACC (Laparoscopic Approach to Cervical Cancer) Trial were presented for the first time at the SGO meeting and were later followed by a second trial presented at the ASCO meeting in June 2018, both studies were shortly afterwards fully published^5^. The LACC trial was a large phase III multicenter RCT with surprising results: the minimally invasive radical hysterectomy was associated with lower rates of disease-free survival and overall survival than open abdominal radical hysterectomy. Furthermore, the rate of local recurrence and distant metastases was also higher in the MIS group. And of note, there were no significant differences in QoL measurements and complications, which could be demonstrated in two further publications^5–7^. A second analysis (US epidemiological database, SEER Database) in 2018 from Melamed et al. also showed that minimally invasive radical hysterectomy was associated with shorter overall survival than open surgery among women with stage IA2 or IB1 cervical carcinoma^8^. As a consequence, many centers questioned and already changed the standard of care. However, there were also huge controversies on the published results and their possible implications, reflected by several statements and recommendations of several working groups in Gynecological Oncology, e.g., German Working Group Gynecological Oncology (AGO) and the German Society for Gynecology and Obstetrics (DGGG) and also by the European Society for Gynecological Oncology^9,10^. However, little is known how those results were transformed into everyday clinical routine.

Therefore, the NOGGO and AGE conducted a nationwide survey in Germany to get first insights shortly after full text publication of the abovementioned trial. The primary objective of this survey was to get information on the current practice of surgical management of early stage cervical cancer in Germany. Secondary objectives were the reception of the LACC trial and their implications on everyday clinical routine.

## Material and methods

In November 2018 the North Eastern Society of Gynecological Oncology (NOGGO) and the Working Group Gynecological Endoscopy (Arbeitsgemeinschaft Gynäkologische Endoskopie, AGE) initiated a German nationwide survey among gynecological departments on the current standard surgical approach in early stage cervical cancer. Therefore, a standardized digital questionnaire was sent out to 200 gynecological departments in Germany via Email. The North Eastern Society for Gynecological Oncology is one of largest working and study group in Gynecological Oncology in Germany and have a list of all centers participating in trials and who are active members of the society. All of those registered centers were contacted to fulfill the survey. The survey was uploaded on SurveyMonkey. Four weeks after the initial invitation a reminder was sent out to the departments. Study inclusion was closed on 28th of February 2019. The invitation letter included background information and a short summary of the results of the LACC Trial and also a link to the original Publication by Ramirez et al. Only one questionnaire could be filled in by each department.

The questionnaire was divided into 4 sections. Question Format was either yes/no or categorized in four to five possible answers, also free text answers were in some questions possible. The first section contained baseline sociodemographic information but also included questions on centers´ expertise and numbers of cervical cancer cases per year (primary diagnosis, radical hysterectomies). The second section asked after the current surgical approach (minimally invasive, open abdominal, vaginal, robotic or combined surgery) of each department regarding the type of classification of radical hysterectomies and the indication for the latter (according to FIGO Classification). The third part of the questionnaire consisted of specific questions on the LACC Trial: Were the centers aware of the published results (e.g. survival outcomes, QoL, surgical outcomes) and how did they get to know about the quality and reliability of the results? What are possible explanations and reasons of the (partly) unexpected results?

The last section yielded on consequences of the reported results, most importantly whether the standard care has already been changed and what possible reasons were not to change them. Furthermore, the centers were asked on their opinion on the need of further trials on the surgical approach in cervical cancer overall and specifically in Germany.

Descriptive Analysis was performed as well as logistic regression analysis. For statistical analysis IBM SPSS Statistics Version 25 was used.

Informed written consent was given by all of the participants. Furthermore the survey was approved by the local IRB (Charité University Hospital Berlin, Germany), however an ethical approval was not necessary according to local regulations.

### Ethical approval

This article does not contain any studies with human participants or animals performed by any of the authors.

## Results

Altogether 186 centers completed the survey. 9 questionnaires had to be excluded (double answering) and one questionnaire did not contain any information, so 176 questionnaires were included in the final analysis. Median age of the respondents was 50 years with a range of 31–74 years and 25th and 75th percentiles of 40 and 56 years, respectively. Median years of professional experience as physicians was 22 years (range of 4–40 years, quartiles of 13 and 30 years). Seventy-one percent were specialists in gynaecological oncology (n = 126), 22% in general oncology (n = 39), 20% in obstetrics/feto-maternal medicine (n = 35) and the remaining 23% did not have a specialization or not yet (n = 40), double answers were possible in this question. When asked whether the study participants had ever received a certificate of the German society of minimally invasive surgery (MIC Level I, II or III) and thereby qualify themselves as experts in laparoscopy, 93 respondents stated to have achieved such a certificate (53.1%) whereas 82 did not (47.9%). However, there is no published verification of those defined Levels of expertise.

Seventy-two percent of all respondents were male (n = 127) and 28% female (n = 49). About half of all (53%) were head of their departments, another 42% had a leading position and the remaining 4% were just specialists in gynecology. Twenty-one percent worked for a university hospital (n = 37), 29% for a full-service or tertiary care hospital (N = 52), 21% for a local hospital (n = 37) and 18% for a private hospital (n = 31).

Over two thirds of the centers had already been taken part in surgical clinical trials (yes = 67%, no 33%). Overall active participation in clinical trials was answered with yes by 82% of the centers, which means that they were actively recruiting patients in trials. Almost two-third were certified centers for gynecological oncology according the Certification process and guidelines of the German Cancer Society, whereas 37% did not have such a certificate. Detailed numbers can be found in Table 1.

**Table 1:** Detailed information about the expertise of the participating Centers and Hospitals.

	Yes	No
Aware of LACC results	92%	8%
Attending in clinical trials (surgical/non surgical)	67%/82%	33%/18%
Certified Breast Cancer Center	78%	23%
Super specialisation in Gynecological Oncology	71%	29%
Certified Gynecological Cancer Center	63%	37%
Certificate for Minimal invasive Surgery (> MIC I)	39%	61%
Type of Hospital	University Hospital 21% /Maximum care 29%	Municipal 21%, private 18%
Current position	Head of Dept. 51% Senior Consultant 41% Junior Consultant 8%	

The median number of cervical cancer cases treated per year was 15 (max 120, min 0), whereas the median number of treated gynecological malignancies was 93.

Overall, the number of “simple” hysterectomies performed in each center was 180. Preferred surgical technique in cervical cancer was interestingly per laparotomy (54%) over laparoscopic (34%), vaginal (8%) or robotic approaches (5%).

The majority of the participants (92.5%) already knew the results of the LACC Trial, whereas only 56% knew in November 2018 about the results of the SEER database analysis. Also, 67% of the asked German Centers were surprised by the published results regarding PFS and OS and likewise did not expected the complication rate in the MIS versus open surgery groups. Quality rating of the LACC-Trial was overall good to very good, only 1.3% rated the quality as very bad. Furthermore, for over half of the centers the LACC trial results are also applicable and transferable to Germany. However, from 176 Departments in Germany 89 answered that according to the LACC trial results the standard of care has been changed in their Centers (60%) although a higher percentage (83%) was aware of a German national guideline published by the German Society of Gynecology and Obstetrics (DGGG) recommendation of changing the standard.

**Figure 1:**
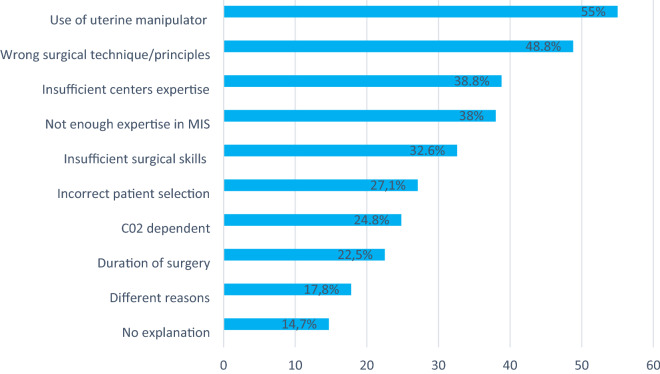
Reasons (according to given possible answers in the survey) for inferior outcome in the MIS group.

Centers were also asked for possible reasons and explanations for the inferior outcome of the MIS group in the LACC trial. Results in the present survey were inhomogeneous, detailed information can be found in Fig. 1. Mainly wrong surgical technique and the use of a uterine manipulator were seen as the main reasons.

The non-favorable outcome for OS and PFS in the MIS arm was not detected for larger tumors in the LACC trial and SEER database. However, for the 176 participants of this survey the reason for this was in the majority a higher risk of lymphogenic metastasis in larger tumors (50%) and “easier” or different applied surgical techniques in earlier stages (59%).

However, a significantly lesser percentage was willing to change their standard of care. To better identify possible influencing factors yielding to that decision and to better distinguish between groups favoring the surgical standard of MIS instead a recommended change to open surgery several logistic regression analyses were performed. The present results show that certified centers for gynecological oncology, centers who are actively participating in clinical trials and centers with larger volume (cervical cancer cases per year) were more likely to change surgical treatment of early stage cervical cancer to open surgery *(*p < 0.001 and p = 0.021respectively). Also Centers which rated the trial´s quality higher and rated the results as transferable to Germany were also more willing to change. We also tried to evaluate the differences between centers who did not change yet but tend to. Results could demonstrate that the volume of the center regarding number of “simple” hysterectomies (more than 100 or less than 100) was significantly associated with the willingness of changing the routine (p = 0.001).

## Discussion

Laparoscopic or minimal invasive surgery has been the standard of care in the treatment of early stage cervical cancer. Several techniques have evolved over the past decades^11^. In many cases techniques were redefined with regard to new anatomical and functional understandings of radical hysterectomy and yielding also to reduce surgery associated morbidity and improve QoL parameters^12,13^. However, the recently published LACC trial were surprising and contrary to the outcomes in various other retrospective studies which compared outcomes in MIS and open arms for carcinoma cervix^14–17^. Interestingly the trial was planned as a non-inferiority design but failed to achieve the primary study outcome. Moreover, after the trial was enrolled in June 2008, it had to be stopped accrual prematurely in June 2017 when safety concerns were raised by the Independent Data Safety and Monitoring Committee when there was an apparent inferiority of MIS arm. However, the LACC trial and further analysis could demonstrate poor outcome for MIS in early stage cervical cancer^18–20^. This was significant not only for OS but also for local recurrence and distant metastasis. There were several comments^21^ and controversies after full text publication the trials by Ramirez et al. and Melamed et al., nonetheless there were also many recommendations among international societies to change standard of care. Also in Germany the leading study and guideline groups commented on the trial^22^. However, the present survey represents some first “real life” insights into the perception of the LACC trial results and represents a large majority of Centers among Germany treating (early stage) cervical cancer. Centers were of high expertise and high volume and did, not surprisingly, already knew about the results of the published trials and were surprised by the results. In contrast a significantly lower number of the hospitals would change the standard of surgical care to open surgery. Despite rating the LACC Trial as qualitative “high” to “very high”, there seems a lack of knowledge for possible explanations for the inferior MIS outcome. Internationally the use of uterine manipulator and the lack of surgical skills and/or wrong patient selection were mentioned besides conflicting histopathological results and also very good outcome parameters in the open surgery arm compared to other trials^23^. The results of the present survey fit well in this picture as mainly the same reasons were given by the German centers. However, the LACC trial is a prospectively randomized controlled trial and meets the highest standards of evidence based medicine. It is also so far, the largest RCT addressing this topic worldwide, the findings are also supported by further retrospective and national database analysis. Nonetheless also among the Hospitals interviewed in the present survey by NOGGO and AGE there is a high wish and need for further prospective trials regarding surgical treatment of cervical cancer. Furthermore, there are two more ongoing trials investigating the role of MIS in early cervical cancer survival (NCT03738969 and NCT03719547).

## Conclusion

We conducted a survey study by means of a digital questionnaire about the reception of the LACC trial on surgical treatment of early stage cervical cancer among gynecological oncologists, medical oncologists and experts in minimally invasive gynecological surgery in Germany. Although the LACC trial is widely regarded as a randomized controlled trial with high relevance only half of all study participants expressed their willingness to change their everyday clinical and surgical routine in the treatment of early-stage gynecological cancer. This willingness was significantly associated with higher expertise and the level of active study participation.
